# The role of mechanobiology in bone and cartilage model systems in characterizing initiation and progression of osteoarthritis

**DOI:** 10.1063/5.0068277

**Published:** 2022-01-05

**Authors:** Tom Hodgkinson, Isabel N. Amado, Fergal J. O'Brien, Oran D. Kennedy

**Affiliations:** 1 Department of Anatomy and Regenerative Medicine, Royal College of Surgeons in Ireland, Dublin, Ireland; 2 Advanced Materials Bio-Engineering Research Centre (AMBER), Dublin, Ireland; 3 Trinity Centre for Biomedical Engineering, Trinity College Dublin, Dublin, Ireland

## Abstract

Multifaceted changes in the mechanobiological environment of skeletal joints, at multiple length scales, are central to the development of diseases-like osteoarthritis (OA). Recent evidence demonstrates related mechanical alterations in both bone and cartilage tissues, with crosstalk between the tissues being an important factor in acute and chronic degenerative processes. However, recapitulating multicellular tissue systems in the laboratory to study the entire osteochondral unit remains challenging. Thus, the development of accurate and reproducible OA model systems and the selection of the most suitable model for individual experimental approaches are critical. This review first discusses recent progress in understanding mechanosensory processes in healthy and osteoarthritic joints. Subsequently, we review advancements in the development of *in vitro* and *ex vivo* model systems ranging from 2D monocultures through to joint organ-on-a-chip models. Use of these systems allows for the study of multiple cell types in controlled, reproducible, and dynamic environments, which can incorporate precisely controlled mechanical and biochemical stimuli, and biophysical cues. The way in which these models have, and will continue to, improve our ability to recapitulate complex mechanical/paracrine signaling pathways in osteochondral tissues is then discussed. As the accuracy of model systems advances, they will have a significant impact on both our understanding of the pathobiology of OA and in identifying and screening therapeutic targets to improve treatment of this complex disease.

## INTRODUCTION

The healthy functioning of skeletal joints is often considered in terms of articular cartilage only. However, in addition to cartilage, the subchondral bone, synovial lining, ligaments, and other connective tissues form an integrated system that maintains form and function. Each of these tissues contributes to overall functionality and damage to any of them can impact joint integrity. Considering the joint at this level is crucial to understanding its function as well as its response to injury and disease—all of which are significantly influenced by the mechanical environment. The appendicular joints in the skeleton have evolved to allow almost frictionless articulation while bearing significant mechanical load.^1^ While physiological loading has long been known to be essential for healthy maintenance of joint homeostasis, supraphysiological conditions can drive degradative processes, such as osteoarthritis (OA).

OA is now considered to be a multifactorial disease of the whole joint.^2–4^ In cartilage, a cell-mediated shift from anabolism to catabolism by the resident cells, chondrocytes, drives the degeneration of the extracellular matrix (ECM). In the subchondral bone, dysregulation of bone remodeling and osteophyte formation are also part of the disease process. At present, the timing of, and relationship between, these phenomena from a pathophysiological perspective is not clear.^1^ It is known that the altered structure and composition of joint tissues often suggest that the physical microenvironment of resident cells themselves is changed. However, at a cellular level, understanding the mechanisms by which intrinsic properties of the ECM (e.g., stiffness) and extrinsic forces (e.g., compression/tension etc.) are transduced is non-trivial. Even in a single cell or tissue type (i.e., bone or cartilage) in isolation, the process is not straightforward. In OA, characteristic ECM degradation leads to localized changes in mechanical stress, driving cell stress responses, inflammation, and senescence/apoptosis. Eventually, a feedback loop is established whereby pathological cell phenotypes produce poor-quality ECM. This contributes to the degradation of remaining ECM and so drives further joint destruction.^5^ Thus, despite the diverse origins and complex etiology of OA, it is clear that the disease progression invariably involves multiple joint tissues as well as alterations in their mechanobiological properties.

Studying this multitissue dynamic joint system, and the complex manner in which it changes with injury and disease, remains challenging. Representative laboratory model systems have been used for many years, but a true unifying representative system has proved difficult to achieve.^6^ *In vivo* preclinical animal models provide many advantages over laboratory-based *in vitro* options as they allow the whole joint to be considered in the context of a native tissue and mechanical environment. Despite this, many issues surrounding their use remain; they are complex, costly, and time-consuming and require significant ethical consideration (adherence to 3R's principles). In addition, there are several biological concerns with their use, including differences between the human and animal model immune systems, manner of joint loading, and thus cell signaling pathways as well as mode of initiation of OA.^7^ The development of more accurate models would, therefore, be a significant contribution to this field of research.^8^ Conventional *in vitro* models show some benefits due to their well-established and reproducible methods and relatively user-friendly nature but often fail to recreate accurate cell environments. Recently, an increased appreciation of the importance of cell-level mechanobiology has emerged, and these considerations will likely be central to future developments. Alongside these advancements, the development of *ex vivo* or explant models that accurately recreate elements of OA pathology (in particular, the consideration of multiple tissues), including the mechanical environment, has led to a rapid expansion in their use. Explant models can be beneficial as they recapitulate aspects of *in vivo* conditions in the laboratory without some of the challenges of *in vivo* work. In particular, these systems may have value in short-term studies and drug screening applications. Advances in materials science and regenerative medicine will also likely feature heavily in the development of more complex (and ideally more representative) *in vitro* and *ex vivo* models that attempt to accurately recreate cell and tissue physiochemical microenvironments.^6^ This review will first discuss recent advances in the current understanding of mechanosensory processes in healthy and osteoarthritic joints and advancements made in the development of *in vitro* and *ex vivo* model systems ranging from two-dimensional (2D) monocultures through to joint organ-on-a-chip models. Finally, details of therapeutic targets identified and tested using these models will be discussed.

## BASIC FORM AND FUNCTION OF THE OSTEOCHONDRAL TISSUES

Osteochondral joint tissues experience mechanical stress over a wide range throughout their lifetime.^9^ Bone is a key structural and protective element of the skeleton and is a complex, heterogeneous anisotropic material. The subchondral bone can be subdivided into several components, which combine to perform specific functions.^10,11^ The subchondral plate, directly adjacent to articular cartilage, primarily prevents shear forces from damaging joint tissues by dispersing them as compressive and tensile forces.^12^ Below the subchondral plate, the bone becomes a porous network of trabecular bone in which individual trabeculae are orientated along local “stress-lines,” as described by the “Wolff's law.”^12,13^ This load-sensitive structure is maintained through the coordinated action of resident bone cells: osteoblasts, osteoclasts, and osteocytes. In healthy bone, it is the resident (and most numerous and ubiquitous) osteocytes that “sense” when and where bone tissue needs to be removed.^14–16^ A pro-osteoclastogenic signaling cascade is produced, such that osteoclasts can carry out this task via resorption, and that tissue is then replaced via coupled osteoblast-mediated bone formation.^17^ This process is continuous, dynamic, and is aided and influenced by a range of specialized growth factors and hormones. Multiple mechanosensing mechanisms have been identified in these cells, including membrane channels, integrins, the cytoskeleton, and primary cilia.^18–22^

The structure of articular cartilage is also highly adapted to withstand mechanical loads, which it absorbs and disperses in an almost frictionless environment during movement. Cartilage achieves this through the anisotropic, hierarchical structure, and specialized composition of its ECM. The anisotropy of cartilage can be seen at the ECM level from the joint surface to the subchondral bone in its superficial, middle, and deep/calcified zones, and at the pericellular level (i.e., pericellular, territorial, and inter-territorial ECM).^23,24^ At the joint surface, superficial zone chondrocytes are flattened and aligned parallel to joint surfaces and produce a low-friction matrix rich in hyaluronan and lubricin. Deeper in the tissue, middle zone chondrocytes are larger/rounder, and type II collagen fibrils increase in thickness. Aggrecan is the most abundant hydrophilic proteoglycan in cartilage, and its concentration increases as a function of tissue depth.^2,24–26^ Other strongly hydrophilic proteoglycans and associated glycosaminoglycans allow cartilage to retain large quantities of water, which facilitates tissue resistance to compressive forces and decreased friction during movement. Many of the cellular mechanosensing mechanisms that exist in bone also exist in chondrocytes, including membrane channels, integrin activation, the cytoskeleton, and primary cilia, which have significant effects on anabolic and catabolic cell processes.^27^

During skeletal loading, complex gradients of mechanical stress and biochemical signals are generated that have profound impacts on cellular responses in the joint. The mechanisms of these important processes are yet to be fully understood.^28,29^ Furthermore, during the initiation and progression of injury/disease, it becomes even more important to understand these processes to develop new and more effective treatments.

## MECHANISMS OF MECHANOTRANSDUCTION IN OSTEOCHONDRAL TISSUES

**FIG. 1.**
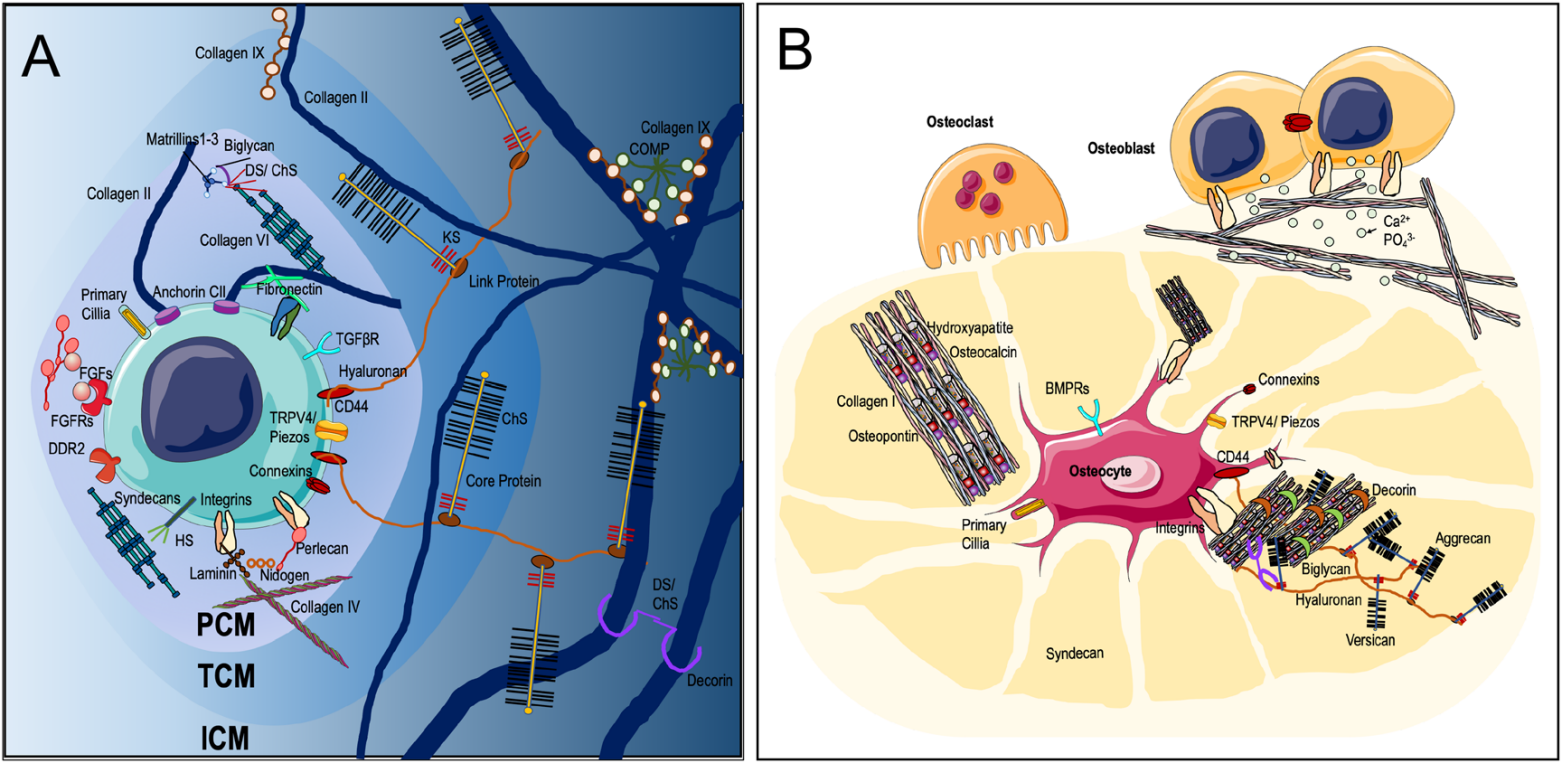
Extracellular matrix environment and mechanosensory mechanisms in (a) cartilage and (b) bone. In cartilage, chondrocytes possess a number of cell surface receptors involved in mechanotransduction including integrins, syndecans CD44, and discoidin domain receptor 2. Mechanosensitive ion channels such as transient receptor potential cation channels, piezo-channels, and connexons. The primary cilium, a mechanosensory organelle, contains high concentrations of mechanosensing machinery central to cell responses to mechanical forces. Chondrocytes are immediately surrounded by the pericellular matrix (PCM), which is characterized by the expression of type VI collagen. The PCM acts as a force transducer for the cell and determines the local mechanical and biochemical environments. Moving out the PCM integrates with the territorial matrix (TCM), which contains fine type II collagen fibers, proteoglycans, and glycosaminoglycans like keratin sulfate (KS), chondroitin sulfate (ChS), heparin sulfate, hyaluronic acid, and aggrecan. The TCM acts with the PCM as a reservoir for growth factors, which are released or presented to cell surface receptors on mechanical deformation. The TCM integrates with the interterritorial matrix (ICM) containing large type II collagen fibers, along with non-fibrillar collagens like type IX collagen, which is associated with cartilage oligomeric matrix protein (COMP). The TCM also contains high levels of hyaluronic acid, aggrecan, and other proteoglycans, which retain high amounts of water within the ECM.

In order for cells in osteochondral tissues to respond to their physical environment, they must have the ability to sense specific parameters of the mechanical loads they sustain, such as its type, duration, and frequency. Multiple sensory mechanisms by which osteochondral cells achieve this have now been identified (Fig. 1) and are reviewed below.

### Mechanotransduction in bone tissue

By virtue of their function, joints experience loading as a complex combination of compression, tension, shear, and hydrostatic and osmotic pressures. At a basic level, compressive forces, generated by body weight, are transmitted through surface cartilage to the subchondral bone and then away from the joint surface.

The relatively high stiffness of the mineralized bone means that strains experienced by resident cells (osteocytes) *in vivo* are significantly lower than in the cartilage-of the order of 0.05% strain during normal activity and 0.2% during strenuous activity.^30^ Some mechanosensory mechanisms are common between bone and cartilage cells, including integrin activation, intracellular kinase cascades [e.g., mitogen-activated protein kinase (MAPK), extracellular signal-regulated protein kinase (ERK1/2)], intracellular calcium release, and membrane channel activation.^31–33^ As discussed above, osteocytes play a dominant role in mechanosensation in bone. Ablation of osteocyte populations significantly increases porosity in cortical bone and decreases overall sensitivity to mechanical loading.^34^ Integrin activation is central to bone cell responses to deformative and non-deformative loadings. In particular, β1 and β3 integrins are important for mechanosensing in osteoblasts and osteocytes (notably the same is true for chondrocytes—detailed below).^35,36^ Mice with osteoblast or osteocyte-specific dominant negative forms of β1 integrin or β1-ablation exhibit reduced bone mass and increased porosity due to increased osteoclast activity.^37,38^ In osteocytes, β1 integrin is preferentially expressed in the cell body, while β3 is predominately expressed along the cell processes.^39,40^ Aside from integrin signaling, hydrostatic pressure and fluid flow are critical mediators of bone remodeling. Hydrostatic pressures over a broad range (∼5 kPa–4 MPa) have been reported to influence bone cell behavior. Dynamic pressures ∼10 kPa have been shown to regulate 3′,5′-cyclic monophosphate (cAMP) and Cyclic guanosine monophosphate (cGMP) cellular accumulation, increase alkaline phosphatase activity, osteogenic gene expression, ECM mineralization, and promote resorptive-like phenotypes in osteoclasts.^41,42^ Dynamic pressures in the ∼30–100 kPa range promote anti-osteoclastogenic phenotypes in bone marrow cells. Even larger dynamic pressures (in the MPa range) have been shown to increase cell–cell and cell-matrix adhesions, promote actin reorganization in the cytoskeleton, and, in bone explant tissues, promote cell viability.^43,44^ Localized load-induced pressure fluctuations also generate low velocity fluid flow through the canicular network, which is now thought to be the primary mechanism by which osteocytes sense mechanical loading. Osteocyte responses to fluid flow require a functioning actin cytoskeleton and involve primary cilia, with loss of the cilia attenuating cell mechanosensitivity. On experiencing fluid flow, osteocyte signaling generally involves release of nitric oxide (NO), Adenosine triphosphate (ATP), Ca^2+^, and activation of ERK1/2, which, in turn, regulates numerous bone remodeling pathways including receptor activator of nuclear factor kappa-B ligand (RANKL) expression, cell proliferation, matrix metalloproteinase-13 (MMP13) expression, and osteogenic differentiation of MSCs.^45–48^ Key differences in osteocyte responses to oscillating and unidirectional flow have been reported.^18,49–54^ For example, unidirectional flow increases intracellular calcium through the release of intracellular stores and membrane channel activation, while no such activation of membrane channels has been reported in cells subjected to oscillatory flows.^50,55^

In addition to being highly mechanosensitive, osteocytes are also expert communicators. Despite being embedded in the mineralized matrix, osteocytes directly communicate with each other and with other local cell-types through gap junctions.^56,57^ This allows for the transmission of information from one part of the matrix to another, thus, in turn, allowing for the regulation of bone formation and turnover.^58^ Furthermore, gap junction phosphorylation and activity are, in part, mechanically regulated,^59^ and in the case of osteoblasts, they have been shown to open and become phosphorylated in response to fluid flow, resulting in increased ATP and prostaglandin release.^59–61^ While the blocking gap junction activity was found to prevent fluid flow-mediated production of osteopontin and osteocalcin.^62^ Taken together, these findings demonstrate that mechanotransduction is a central aspect of healthy bone function.

### Mechanotransduction in cartilage tissue

Since cartilage covers the majority of articulating surfaces, it naturally takes the majority of the load imparted at the joint. At the cell level, chondrocytes perceive load-induced tissue deformation through a secreted pericellular matrix (PCM).^63^ The PCM is characterized by the presence of collagen type VI,^64^ perlecan,^65^ aggrecan,^66^ laminin,^67^ fibronectin,^68^ hyaluronan,^69^ biglycan,^70^ and type IX collagen.^71^ The PCM can modulate mechanical stress, osmotic pressure, and fluid-flow in the chondrocyte microenvironment, thus acting as a key regulator of mechanotransduction.^72^ Though significantly stiffer than the chondrocyte itself (Young's modulus ∼40–100 vs ∼0.5 kPa), the PCM is softer than the cartilage ECM surrounding it (∼0.1–2 MPa).^73–76^ The PCM and Territorial Matrix (TCM) also have a role as natural, mechanoresponsive growth factor reservoirs. For example, TGFβ and FGF bind to heparin sulfate domains in PCM/territorial matrix molecules, such as perlecan, and can be released to activate cell receptors by mechanical deformation.^72,77,78^

Chondrocytes also sense tissue deformation directly through integrin-matrix adhesions. Under physiological load, integrins initiate chondrogenic transcription through several mechanisms including the 3′,5′-cyclic monophosphate (cAMP) signaling cascade^79^ and actin cytoskeletal stress-mediated activation of protein kinase A (PKA).^80^ Subsequent activation of cAMP-response element binding (CREB) protein converges on SOX9 phosphorylation, driving chondrogenic gene expression.^81–83^ Additionally, integrin-associated kinases, such as Proto-oncogene tyrosine-protein kinase Src (SRC) and focal adhesion kinase (FAK), activate chondrogenic gene expression through ERK–MAPK-P38 signaling cascades.^84^ Cell deformation also activates membrane channels [PIEZO and transient receptor potential (TRPV) family], causing a Ca^2+^ influx into the cell.^85^ In particular, TRPV channels appear to have a significant role in physiological loading responses. Loss of TRPV4 leads to disruption of cartilage homeostasis and induction of OA,^86,87^ while *in vitro*, the addition of a TRPV4 agonist increased ECM production.^86^ Signaling downstream of TRPV4 has yet to be fully elucidated, but studies have shown G protein-coupled receptor (GPCR) pathway activation [phosphoinositide 3-kinase/Akt/forkhead box protein O (PI3K/Akt/FOXO)], which is thought to be involved in preventing cartilage damage and the onset of premature hypertrophy.^88–90^ Ca^2+^ influx also triggers ATP release through hemichannels (connexons) in the cell membrane and activates ERK1/2 through the anti-catabolic transactivator CITED2 (cbp/p300-interacting transactivator 2).^91–95^

In cartilage, as in bone, hydrostatic pressure and fluid flow also play a role in homeostasis of the local microenvironment. Due to the high-water content of cartilage, hydrostatic pressure bears approximately 90% of applied loads in the tissue.^96–98^ Pressures generated are generally in the 3–10 MPa range (but can reach 18 MPa at certain sites, such as hip joints). Hydrostatic pressure does not deform the tissue itself, but cell adhesion, in particular through α1β1 integrin, remains important for chondrocyte responses to hydrostatic pressure.^99,100^ Hydrostatic pressure, along with osmotic pressure, has significant effects on the activity of transmembrane ion channels and pumps, inhibiting Na/K pump and Na/K/2Cl transport activity but increasing Na/H pump activity and activating TRPV4.^86,87,101–103^ Pressure also activates the purinergic signaling pathway and drives a Ca^2+^ response through inositol-triphosphate-mediated release from sarcoendoplasmic reticulum stores (SERCs).^104^ Inhibition of SERCs, hemichannels, purine receptors, or extracellular ATP blocks cell responses to hydrostatic pressure.^105,106^ Other pathways involved in responses to hydrostatic pressure include estrogen receptor ERα-mediated activation of c-Jun N-terminal kinases (JNK) and increased transforming growth factor receptor (TGFR)I activation.^107,108^

Again, similar to bone, pressure variations in cartilage also generate interstitial fluid flow within cartilage, influencing chondrocyte biosynthetic activities.^109–111^ Numerous studies have shown that low fluid shear stress (∼2–10 dynes/cm^2^) has a chondroprotective effect and initiates repair mechanisms in chondrocytes, whereas high shear stress (∼10–20 dynes/cm^2^) can induce inflammation, cell death, and cartilage degradation.^112–118^ Chondrocyte responses to shear stress also appear to be time-dependent, with short duration (1–2 h) stimulation reducing catabolic responses while a longer stimulation (3–4 h) does not.^118^ Again in this case, shear stress appears to be detected by the primary cilia,^86,87,119–121^ which trigger mechanotransductive signaling cascades and ionic fluxes,^120^ a number of which converge on ERK1/2 and P38 signaling.^122^ Blocking ERK1/2 and P38 with small molecule inhibitors suppresses shear-related increases in ECM production and remodeling.

### Pathological changes in osteochondral tissue and mechanotransduction in OA

**FIG. 2.**
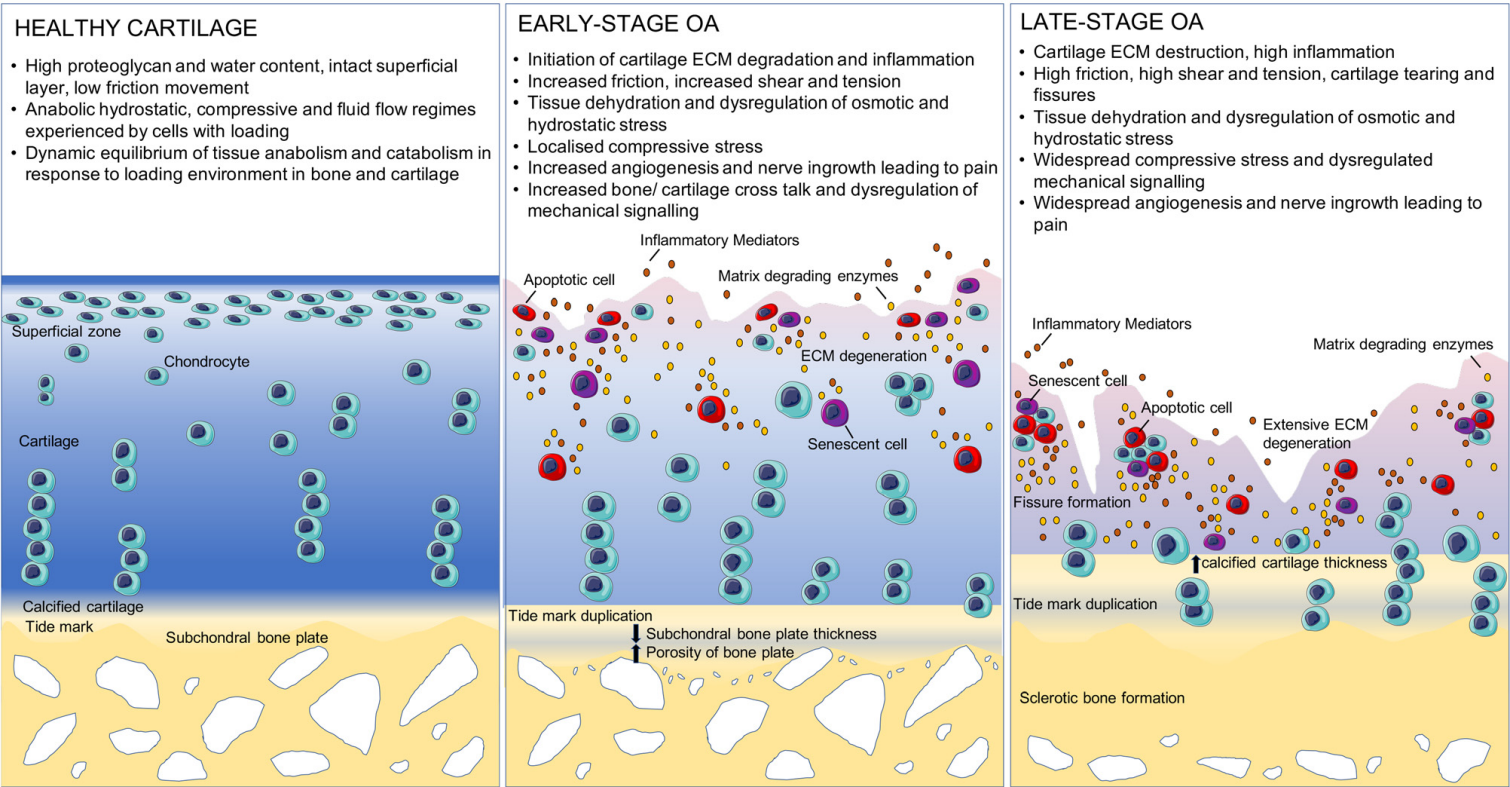
Progression of osteoarthritis in cartilage and bone. In early stage OA, cartilage ECM degeneration by matrix degrading enzymes, such as matrix metalloproteinases, increases frictional, shear, and tensional stress on movement. Changes in cartilage ECM composition decrease tissue hydration, altering fluid flow and hydrostatic and osmotic pressures on loading. The subchondral bone plate decreases in thickness and increases in porosity, facilitating increases in bone-cartilage crosstalk and altering load distribution. These structural changes lead to the development of localized mechanical stress within the tissue, triggering the initiation of catabolic cell responses and mechano-inflammation. Senescent and apoptotic cells secrete disease-propagating molecules [senescence-associated secretory phenotype (SASP)]. The subchondral bone and calcified cartilage also become increasingly innervated and vascularized, contributing significantly to pain development and progression of inflammation. In late-stage OA, high friction, pathological ECM composition, and dehydration drive fissure cartilage fissure formation. Aberrant hypertrophic chondrocyte phenotypes are observed, and the calcified cartilage thickness increases. Sclerotic bone formation thickening of the subchondral bone plate occurs. Mechanical functionality of the joint is compromised, and inflammation and SASP-related secretion drive end stage degeneration and joint pain.

Subchondral bone and cartilage undergo degenerative changes during OA progression (Fig. 2). One of the most pressing questions in the field is whether changes in the subchondral bone occur before or after cartilage changes, and whether changes in the two tissues are causally linked, or independently changing in parallel. Recent clinical studies have suggested that in fact, bone remodeling and composition changes occur prior to detectable changes in cartilage.^123,124^ In the case of post-traumatic OA (PTOA), in the initial aftermath of injury, a transient loss in subchondral bone has been observed through increased osteoclast activity. This period is then followed by an increase in bone formation, density, and volume as OA becomes more advanced.^125–128^ Interestingly, the bone formed in that period is often of poor quality, consistent with the idea that this is a rapid injury-response. Similarly, osteophyte formation occurs at the joint margins in later stages of disease, which causes pain and discomfort. These subchondral changes have long been hypothesized to play roles in disease progression through both biomechanical means and also biochemical bone-cartilage crosstalk.^129,130^ However, the precise mechanisms driving these processes remain elusive. It has long been known that subchondral porosities allow mass transport between bone and cartilage compartments in both healthy and OA joints.^131–133^ Increased osteoclastic activity is thought to increase plate porosity, at least in the initial stages of OA, facilitating crosstalk.^125^ This increased porosity is also linked to vascular invasion of the deep cartilage and aberrant chondrocyte hypertrophy.^134–139^ Studies using murine OA models support this idea, with both cartilage damage and vascular invasion coinciding with increased subchondral porosity and increasing in exchange of soluble factors.^134,140^ In addition to porosity, there is evidence that repetitive loading of joints creates subchondral bone microcracks, even in healthy joints.^141,142^ These microcracks also drive targeted remodeling, through localized osteocytes production of RANKL and decreased osteoprotegerin production.^15,143,144^ Further study is required, however, to understand these acute and chronic responses. As mentioned above, OA-associated accelerated bone remodeling is also connected to lower bone mineralization. OA osteoblasts appear to generate abnormal type I collagen, producing a homotrimer composed of α1 chains rather than the healthy heterotrimer formed from two α1 chains and one α2 chain.^145,146^ This abnormal collagen I production may contribute to deficient matrix mineralization.^12^ In addition to this, OA osteoblasts produce increased interleukin-6 (IL-6), prostaglandin E_2_ (PGE), and TGFβ; the latter drives increased Dickkopf-related protein 2, an inhibitor of mineralization.^12,146,147^ The significance of this shift in bone composition and structure to the overall disease progression needs further exploration. This deficient mineralization is continued as OA progresses and bone deposition and thickness increase, resulting in more bone being present in the area (sclerosis), but reduced subchondral ECM stiffness.^148–151^ Other animal and human studies have examined the effects of inhibiting bone remodeling on OA initiation and progression through the use of bisphosphonates.^152–157^ In these models, bisphosphonate treatment inhibits bone remodeling and attenuates degeneration of cartilage. However, to date in the clinic, while bisphosphonate treatment has been shown to be effective at inhibiting bone remodeling, they have not shown conclusive effects on modulating cartilage degeneration in OA patients.

Osteocyte density and morphology are also altered in the subchondral bone of OA joints. The number of viable femoral head osteocytes is decreased in OA, while their markers are dysregulated, and cell–cell communication via gap junctions is decreased. These changes correspond to an increase in new bone formation and total bone volume, although as above—the mineral content and quality are altered.^158–160^ Recent reports have highlighted a fascinating link between OA and dysregulation of osteocyte remodeling of their perilacunar/canalicular channels (PLR).^161–165^ In these studies, MMP13 was selectively ablated in murine osteocytes, but not in chondrocytes.^161^ Not only these mice suppressed PLR in cortical and subchondral bone, but these changes also significantly impacted cartilage, reducing proteoglycan content, altering the production of type II collagen, aggrecan, and MMP13, and increasing the incidence of cartilage lesions. All of which are consistent with the development of early OA. These findings highlight a role for osteocyte-cartilage crosstalk, and in particular, a causal role for suppressed PLR in onset of OA.

In cartilage, OA-related degradation of the hydrated, proteoglycan-rich cartilage matrix leads to macroscopic disruption in the form of fissuring, and chondral flaps or tears.^26,166^ At the cellular level, PCM degradation is an early event during OA progression and has a significant impact on the mechanical environment of chondrocytes.^167^ Recent work showed that preventing this PCM degradation is sufficient to modulate overall disease progression.^168^ Several studies suggest that PCM degradation during OA leads to modulus reductions of between 30% and 50% and increased intracellular Ca^2+^ signaling.^169,170^ These changes in pericellular environment have been linked to cell organization changes observed in OA, whereby healthy columns of cells become disorganized clusters.^171–174^ Recently, this disorganization was used to categorize OA degradation, and chondrocyte PCM stiffness was measured at each stage. Strikingly, significant decreases in PCM stiffness were found between each stage of cellular disorganization and by extension OA progression.^171^ Intriguingly, the complete loss of PCM has been associated with the appearance of long cytoplasmic processes (>8 *μ*m) on chondrocytes, which extend into the territorial ECM.^175,176^ Aside from the direct mechanical implications of decreased PCM stiffness, recent work indicates that such changes are also important in cellular responses to biochemical signaling. PCM degradation also increases chondrocyte exposure to abnormal ECM adhesion sites, specifically type II collagen fibrils (which are more highly expressed in the surrounding ECM rather than the healthy PCM). It is currently thought that this increased interaction, most notably through discoidin domain receptor 2 (DDR2), has significant effects on the cell metabolic process and cell signaling downstream of the receptor,^177,178^ including the upregulation of MMP13 expression.^179,180^

When joint injury or disease causes the development of an abnormal mechanical environment, chondrocytes receive damaging mechanical stimuli and driving catabolic and proinflammatory processes. Studies suggest that piezo-channels are activated in response to supraphysiological loading (strains of 13%–45%), leading to pathologically high intracellular Ca^2+^ concentrations and hyperactivation of downstream responses, cell damage, and apoptosis.^181,182^ Inhibition of piezo-channel activity protects chondrocytes during overload and reduces subsequent cell death.^85^ Despite its role in healthy responses to loading, recent work has shown that under pathological loading, TRPV4 can also trigger apoptotic responses in chondrocytes.^183^ Intracellularly, the transforming growth factor-β-activated kinase 1 (TAK1)-JNK2 cascade is critical to injury responses, acting as an upstream regulator and driving the expression of inflammatory markers and matrix catabolism.^184,185^

## *IN VITRO* MODELS TO STUDY OA AND OSTEOCHONDRAL MECHANOBIOLOGY

Understanding these complex processes in both bone and cartilage tissues individually and in the complete osteochondral unit requires novel experimental approaches and/or a combination of *in vitro*, *ex vivo*, and *in vivo* models. In the sections below, we discuss current experimental models and recent developments, which will help researchers in this field unravel the mechanisms of this condition.

### Two-dimensional (2D) culture systems to study osteochondral mechanobiology

#### Single cell type model systems

**FIG. 3.**
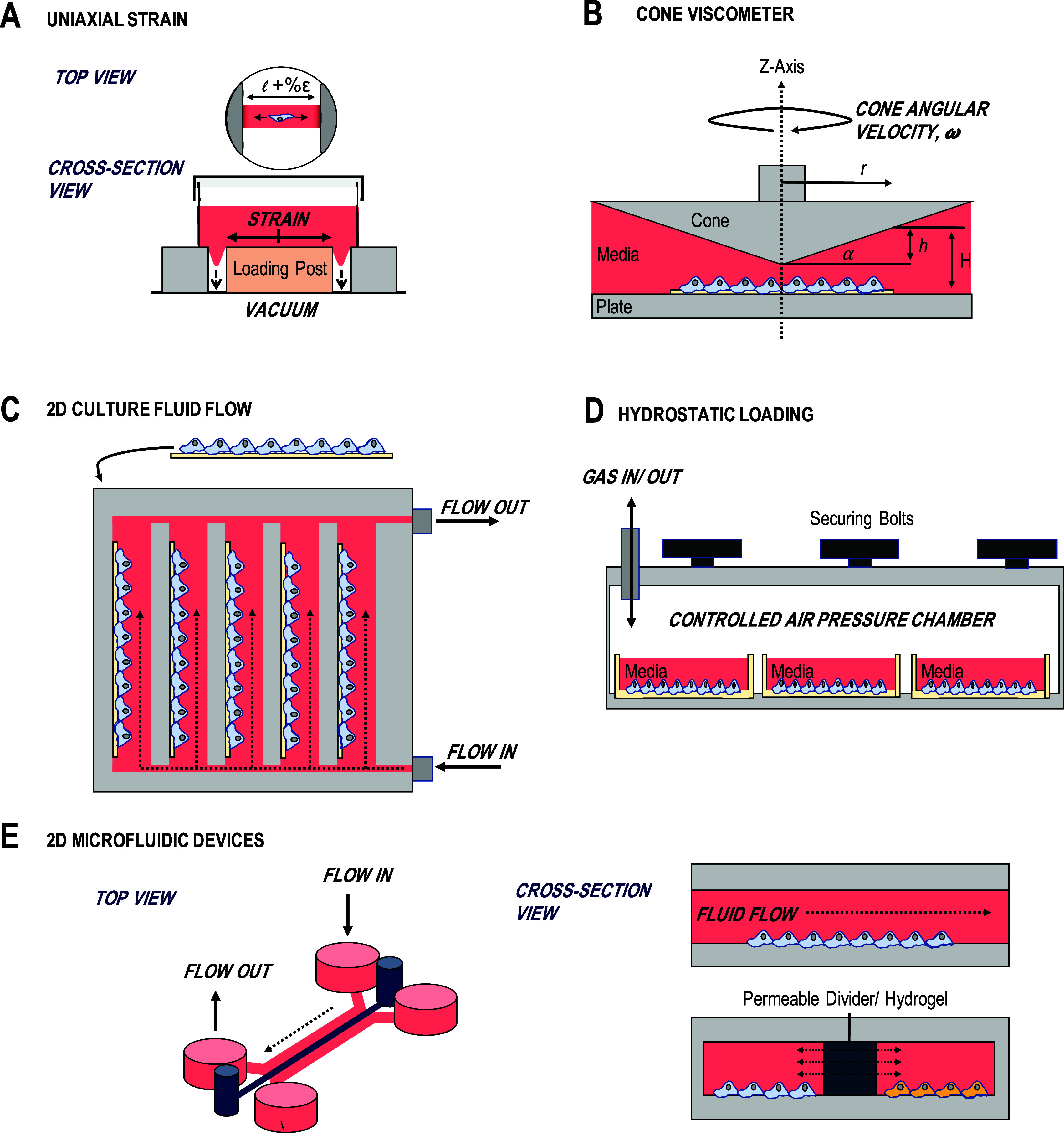
Two-dimensional culture models to study mechanotransduction in osteochondral cells. (a) Uniaxial strain can be applied to cells in monolayer culture by seeding onto deformable membranes. In a typical setup, a vacuum can be applied pulling a polymer membrane uniformly around a loading post and creating a uniaxial strain in the stretched membrane. (b) Simple two-dimensional fluid flow experiments can be conducted in cone viscometer culture, which allows accurate establishment of defined fluid flow across the culture substrate. (c) Fluid flow chambers allow analysis of fluid flow across multiple substrates, which can include different cell types and culture setups simultaneously. Due to directional fluid flow, crosstalk between culture substrates may be limited. (d) Hydrostatic pressure can be applied to cultures through control of culture chamber pressurization. These systems can be used to apply constant or oscillatory hydrostatic pressures to cells in culture over relevant physiological and pathological ranges. (e) Microfluidic devices offer diverse options for steady state and oscillatory two-dimensional culture fluid flow analysis. Alongside this, through the chip design of culture chambers and channels, co-cultures and varied conditions can be achieved simultaneously to recreate *in vivo* conditions and probe pathology relevant signaling.

Two-dimensional culture systems are widely used to examine cell signaling, responses to stimuli, and screen therapeutics. Often, these cell culture models consider single cell populations in isolation (chondrocytes for cartilage; osteoblasts, osteocytes, and osteoclasts for bone). These approaches have utility for studying cell-level responses to stimuli due to the ability to tightly control experimental conditions, rapidly screen multiple experimental parameters and their relative low cost. In the context of the joint, 2D culture systems are commonly used to model OA environments through the supplementation of culture media with proinflammatory cytokines, such as IL-1β and tumor necrosis factor (TNF)-α. The simplicity of such approaches has allowed interrogation of many of the individual intracellular signaling pathways now known to be critical in joint anabolic and catabolic processes. Despite these benefits, these conventional approaches often neglect the significant impact of mechanical stimuli and environment in the osteochondral cell function. Therefore, in recent years, these models have progressed beyond static culture on stiff tissue culture plastic to allow investigators to control the cellular mechanical, along with the biochemical, culture environment. These include 2D hydrogels controlling stiffness and adhesion motifs (i.e., cells seeded onto hydrogel substrates), dynamic substrate tension/cell stretching systems, and systems allowing the generation of fluid flow/shear stress and hydrostatic/dynamic pressures (Fig. 3).^186–193^

One simple system that delivers mechanical stimulation to 2D cell cultures involves seeding cells onto pneumatically or electromagnetically deformed membranes, which can deliver predetermined levels of strain over the designated culture surface. Chondrocytes stimulated in this way with physiological-like tensile strain (0.5 Hz, 10% strain for a duration of 24 h) decrease the expression of catabolic enzymes and increase the expression of anabolic markers such as aggrecan.^194–196^ Larger strains and durations were shown to have an opposing effect, increasing catabolic MMP-1, -3, -9, and -13 expression.^195–197^ Similar studies using 2D membrane-stretching systems seeded with bone cells identified signaling pathways involved in responses to stretch, which are directly translatable to more complex models and even the *in vivo* environment. For example, in osteocytes, cell stretching (5% elongation over 1–20 min) has an anti-apoptotic effects through ERK1/2 activation.^198^ In osteoblasts, numerous studies applying cyclical stretching have shown that osteoblastic maturation is accelerated, osteogenic gene expression is increased, and ECM deposition enhanced,^199–201^ while in osteoclasts, bone resorbing activity is increased by cyclic stretching in 2D cultures.^202^ These devices have been limited by their relatively low through-put capabilities. However, ongoing research is significantly increasing the throughput of these types of stretching systems.^203,204^

Aside from cell stretching, fluid flow systems allow the investigation of the effects of shear on osteochondral cells. These typically involve application of controlled unidirectional or oscillatory fluid movement in a cell culture chamber and range from rocking cultures and cone viscometers to complex microfluidic devices. In chondrocytes, cone viscometer experiments have helped to define flow conditions that elicit catabolic or anabolic responses and study morphological and molecular responses. For example, continuous, unidirectional laminar fluid flow (1.64 Pa) was found to significantly decrease the expression of type II collagen and aggrecan and increase nitrite levels in culture media, indicating cell stress.^205^ Higher shear stress regimes (3.5 Pa for 4 days) result in rounded chondrocyte morphology in comparison to static cultures,^206^ while shear stresses of 2 Pa were found to regulate IL-6, toll-like receptor (TLR)4, and caveolin-1 synthesis in a cyclooxygenase-2 (COX-2)-dependent manner.^207^ Similarly, the effects of fluid flow on bone cells have been studied in a variety of experimental systems.^208,209^ For example, in a parallel plate chamber, oscillatory flow was found to increase osteoblastic marker gene expression and modulate the activity of alkaline phosphatase (ALP) in mesenchymal stem cells (MSCs).^210–212^ Pairing these mechanostimulatory techniques with -omics analysis provides valuable insights into cell responses to similar stresses that might occur *in vivo*. For example, pairing oscillating fluid flow with transcriptomic microarray analysis in osteocyte-like cells (MLO-Y4) demonstrated that at 1 Pa peak shear stress for 2 h, ATP producing enzyme nucleoside-diphosphate kinase (NDK), calcium-binding calcyclin, and G-protein couple kinase 6 were all significantly upregulated.^213^ Such investigation has indicated that bone cells respond differentially to oscillatory and steady flow, with oscillatory flow conditions being advantageous for bone formation *in vitro.*^212,214,215^

Hydrostatic pressure can also be simulated in culture to study osteochondral cell responses. Applying intermittent hydrostatic pressure over a range (1–4 Hz, at 1, 5, and 10 MPa for 4 h per day for 4 days) through hydraulic loading was found to alter healthy and OA human chondrocyte phenotypes.^216^ Application of higher pressures (5 and 10 MPa) corresponded with the upregulation of aggrecan and type II collagen at gene and protein levels.^216^ This experimental approach also allows the simultaneous study of the effects of mechanical and biochemical stimuli by supplementation of culture media. For example, in a separate study, bone morphogenetic protein (BMP)-2 was applied to human OA chondrocytes, with and without the application of hydrostatic pressure (10 MPa, 1 Hz, 4 h a day for 4 days). Through this work, it was demonstrated that the growth factor and mechanical stimuli had complementary effects. BMP-2 was found to increase aggrecan, but when applied with hydrostatic pressure, an increase in type II collagen was also observed. The expression of the catabolic marker MMP-2 was also decreased with hydrostatic pressure application but not when BMP-2 was supplied alone.^217^ These results highlight how these systems can further our understanding of the mechanical and biochemical interplay required for healthy anabolic chondrocyte phenotypes and also the fine balance that exists between different stimuli. Interestingly, while physiological levels of hydrostatic pressure (5–10 MPa) have been shown to decrease catabolic marker genes like MMP-13 and a disintegrin and metalloproteinase with thrombospondin motifs 5 (ADAMTS5), high hydrostatic pressures in the range of 50 MPa (replicating joint overloading) have been shown to increase the expression of vascular endothelial growth factor (VEGF), which could be one of the mechanisms by which vessel invasion of the cartilage is increased in OA.^218–220^ Interstitial fluid flow in bone through the lacunar-canalicular system is important to maintain bone homeostasis. Similar to work with chondrocytes, simple cyclic hydrostatic pressure bioreactors can be used to investigate bone cell responses. Using such approaches, cyclic hydrostatic pressures in the range of 10–300 kPa (0.5–2 Hz) have been found to increase the expression of osteogenic markers like RUNX Family Transcription factor 2 (RUNX2) and osteopontin in MSCs.^221^ *In vivo*, the mode of mechanical stimulation may impact cell responses in a cell-type dependent manner. Comparisons of the effects of fluid flow and hydrostatic pressure on osteoblastic MC3T3 cells showed that the two modes of mechanical stimulation had differing effects, for example, fluid flow has increased ATP release and F-actin fiber formation, while hydrostatic pressure did not, despite both increasing COX-2 expression.^222^ This further highlights the need for careful selection of the most relevant mechanical stimuli for the investigation of specific signaling responses.

#### Co-culture model systems

2D co-culture systems allow for the investigation of multiple cell types cultured in shared environments and can help determine the complex bone-cartilage signaling crosstalk processes in osteochondral tissues. Critical parameters for co-cultures include the type of cells, culture media, order in which cells are cultured, and numbers/ratio of cell types. Even though optimization of these technical details can be time consuming, such co-cultures are valuable tools to systematically increase culture complexity and move closer to recapitulating real tissue microenvironments while retaining control over experimental conditions.^223^ These systems can involve direct cell–cell contact or cells not in direct contact but sharing culture environments, for example, through the use of cell culture inserts or microfluidic chambers. Such systems have been employed to determine, for example, that co-culturewith chondrocytes can improve the chondrogenic differentiation of MSCs through mechanisms involving both soluble factor secretion and cell–cell contact.^224–226^ As with monolayer cultures, combining these co-cultures with methods to mechanically stimulate the cells will provide new insights into their function and behavior. For example, combining tensile stimulation with co-cultures of MSCs and chondrocytes was reported to increase chondrogenic phenotypes and rapidity of cell expansion.^227^ Though few reports of 2D osteochondral co-cultures under tensile stimulation exist, the examination of fluid shear on cell–cell communication is a promising area of research, particularly through microfluidics. Microfluidic systems can be used to create complex multicompartment co-cultures with precise control of fluid flow and physical parameters, while the integration of sensors allows direct read-outs of cell responses.^228^ These microfluidic approaches have been used to show crosstalk between bone cells in response to fluid flow. For example, osteocyte-like MLO-Y4 cells were cultured with osteoclasts (RAW264.7) and exposed to 0.5 Pa shear stress simulation, which resulted in a decrease in RANKL expression in osteocytes, which suggests a reduced osteoclastogenic phenotype.^229^

Though these co-cultures are useful for examining cell–cell interactions, particularly in response to stimuli, the limitations of 2D cultures still apply, not least in terms of altered cell morphologies, a lack of physiological ECM, and cell–ECM interactions.^192^ The use of 2D models in OA research has been hugely beneficial and has provided many of the major steps forward in this field of research as well as establishing fundamental tenets of disease. Nonetheless, some aspects of disease, which are now recognized as being particularly important, cannot easily be captured by these methods.

### Three-dimensional (3D) model systems to study cartilage and bone mechanobiology

Three-dimensional culture systems can recapitulate *in vivo* environments for cartilage and bone cells, where they are surrounded by and interact with ECM. *In vitro* 3D culture models have been developed using a number of biomaterial formats and from a range of naturally and synthetically derived polymers. The most prevalent material formats include lyophilized polymer scaffolds and hydrogels.^230^ These have been produced from natural polymers such as collagen (including gelatin), hyaluronic acid, chondroitin sulfate and alginate, or synthetic polymers, including polyethylene glycol, poly-D,L-lactic acid (PDLLA), and poly(N-isopropylacrylamide (PNIPAM). These polymers confer mechanical and biological properties to materials fabricated from them, which can be tuned to provide cues that can control specific cell responses or mimic the properties of the natural ECM. Natural, ECM-derived materials may also have the added advantage of containing cell attachment motifs as well as an inherent bioactivity that can be cell-instructive. Though alone materials fabricated from these natural polymers may not have sufficient mechanical properties (or range of mechanical properties achievable) for models of the load-bearing joint tissues, they can be easily modified chemically to achieve this. One example of this is gelatin methacryloyl (GelMA), which has been shown to be biocompatible, bioactive, and non-immunogenic.^231^ Other native osteochondral ECM constituents such as hyaluronic acid and chondroitin sulfate are commonly incorporated into materials for both their mechanical and biochemical properties and can be similarly modified chemically to further control their properties.^232^ Though numerous material formats are available, hydrogel models, in particular, allow tight control of 3D cell environments *in vitro* facilitating the investigation of the effects of altering intrinsic matrix microenvironment cues (e.g., mechanical and viscoelastic properties, cell adhesion motif density and type, and degradation) and extrinsic mechanical forces (e.g., compression and tension). Through such investigation, more accurate tissue mimics can be produced to create bone cartilage and osteochondral models.

Changes in ECM properties, including stiffness/strength, significantly impact OA pathogenesis and progression. Within cartilage, gradients of stiffness exist in healthy tissue, from the softest superficial zones to the stiffest deep calcified zones. To investigate the impact of ECM stiffness on chondrocyte behavior and function and on the chondrogenesis of MSCs, previous work has tested chondrocyte and chondrogenic MSC responses to 3D hydrogels over a large range of stiffnesses, with wide variation in results. Differences in material composition, cell source, and mechanical testing methods make definitive comparisons difficult. However, in general, the cartilage ECM produced by MSCs and chondrocytes in hydrogels with stiffnesses between approximately 7.5–40 kPa have been reported to be most similar to the hyaline cartilage observed at joint surfaces.^233–239^ Encapsulation in stiffer materials leads to the formation of hypertrophic chondrocyte phenotypes and initiation of osteogenic processes.^237,240,241^ The differential effects of stiffness on chondrogenic and osteogenic differentiation are of particular importance in designing models of the osteochondral interface. For example, recently hydrogels with tunable stiffness gradients were developed using gelatin-PNIPAM hydrogels containing both beta-sheet and amorphous silk nanofiber solutions. Through a combination of cross-linking and electric field alignment, a gradient of stiffness mimicking that of the ECM stiffness from the superficial to the deep cartilage/subchondral bone was produced.^237^ Using this system, MSC chondrogenesis was enhanced in softer regions of the hydrogel, while osteogenic differentiation was favored in the stiffer regions. This and other similar approaches can be used to mimic natural tissue stiffness gradients and produce multiple differentiated cell types from a single seeded population.^242–244^ Until recently, these investigations into intrinsic hydrogel mechanical cues have typically used elastic materials with varied stiffness or disregarded a material's viscous component. Both bone and cartilage ECM are highly viscoelastic, meaning that they display time-dependent deformation in response to an applied force and corresponding recovery time (relaxation time) for the material to return to its original form. The generation of hydrogels that de-couple stiffness and relaxation times allows the investigation of this effect on cell behavior. For example, alginate-poly(ethylene glycol) (PEG) hydrogels with controllable stiffness (∼3 kPa) and variable relaxation times were used to demonstrate that in faster relaxing gels, bovine chondrocytes significantly increased the volume and interconnectivity of the ECM they produced, while slower relaxation times promoted catabolic processes.^245^ This result highlights the importance of considering viscoelasticity when designing materials for osteochondral engineering and also the utility of these 3D culture systems in understanding the fundamental mechanobiology of the cells in the joint.

Aside from the intrinsic material/ECM properties of 3D models, the application of extrinsic forces can be investigated. The type of force, magnitude, and frequency of extrinsically applied mechanical forces have been determined to drive differential responses in bone and cartilage cells through 3D *in vitro* studies, with even nanoscale displacements able to control MSC osteogenic differentiation.^246,247^ These *in vitro* 3D mechanically stimulated assays are valuable tools as they allow precise control of loading type, magnitude, duration, and biochemical conditions, to understand thresholds for anabolic and catabolic cell responses and differences in the cell signaling. These can be taken into account in the development of treatment strategies.

In cartilage model cultures, compression is most commonly applied to simulate joint loading. Through these studies, compressive loading has been shown to modulate chondrocyte phenotypes, ECM biosynthesis, and inflammatory responses. For example, when stimulated with dynamic compression at physiological magnitudes, hydrogel embedded chondrocytes show reduced inflammatory response to exogenous IL-1β stimulation, increased MAPK and TGF-β pathway activities, and increase their proliferative capacity.^186,190^ Similarly, dynamic compression of chondrocytes cultured in PEG hydrogels increased cartilage-specific ECM deposition.^248^ Aside from compression, these hydrogel systems also allow the testing of other modes of mechanical stimulation, such as cyclical shear. Using different modes of mechanical stimulation has potential to be used to produce cartilage zone specific phenotypic responses or ECM organization in tissue engineered constructs.^249^

#### Co-culture and organ-on-a-chip model systems

**FIG. 4.**
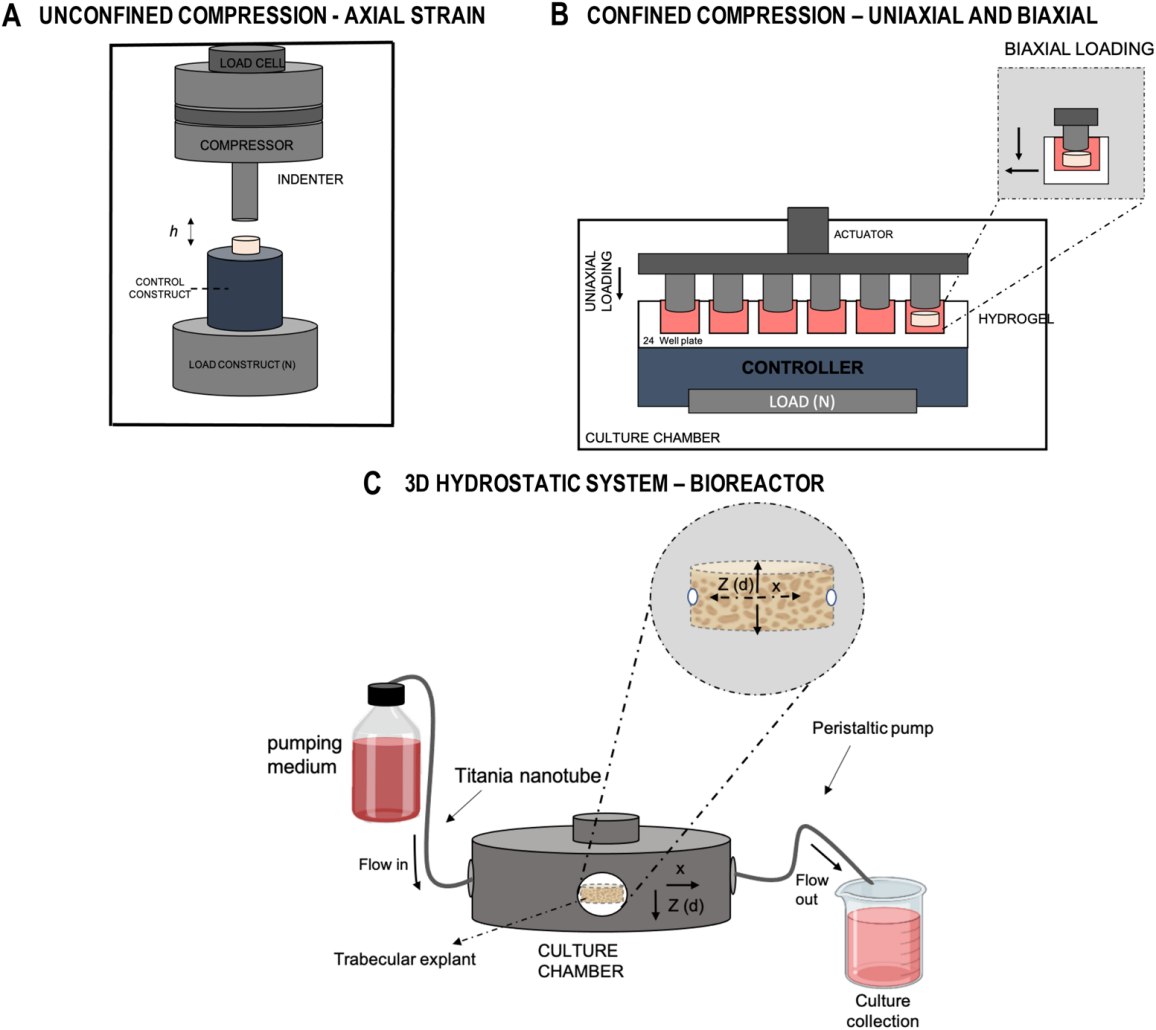
Three-dimensional models to study mechanotransduction in osteochondral tissue. (a) Unconfined compression—axial strain. Compression can be applied to 3D models in culture in unconfined (a) and confined (b) systems. For unconfined compression, mechanical testing machines can be used to apply defined deformation with defined parameters, though often these are limited by single sample analysis. Confined dynamic compression can be applied as a uniaxial or biaxial deformation to a three-dimensional construct. These systems can be applied with standard culture ware to increase the throughput of analysis. For biaxial deformation, simultaneous movement of sliding compartments can be used to result in shear deformation. (c) Schematic of a three-dimensional bioreactor to study bone *ex vivo* explants or scaffold cultures. A hydrostatic pressure bioreactor chamber is used to enable cyclic or continuous mechanical loading through a pumped system to control fluid flow of medium. Z (d) and y represent the direction of the strain created through the trabecular explant.

The next generation of advanced 3D culture systems have the capacity to recreate more complex, joint mimicking environments by supporting multiple cell types or, for example, by directing tissue-specific differentiation MSCs or induced pluripotent stem cells (iPSCs) via controlled physical and biochemical environments^250–252^ (Fig. 4). Creating realistic bone and cartilage environments facilitates the investigation of crosstalk between the two. 3D co-culture models combining osteoblast culture with alginate bead-embedded chondrocytes were shown to be effective at directing bilateral phenotypic thorough such paracrine interactions. Notably, co-culture increased chondrocyte hypertrophy and matrix mineralization. Similarly, a 3D co-culture model system replicating cell–cell interaction between osteoblasts and chondrocytes in the presence of pulsate cyclic tensile stress (15 kPa; 23% strain) reported bilateral phenotypic change with increased chondrocyte hypertrophy, and down regulation of type II collagen, aggrecan, cartilage oligomeric matrix protein precursor (COMP), and SOX-9 expression.^250^ Together, these studies suggest that perhaps through positioning osteoblast and chondrocyte cell populations correctly in models, tissue-like gradients can be created *in vitro*. In a separate study, osteocytes and osteoblasts were seeded in type I collagen hydrogels and stimulated with mechanical loading, which increased type I collagen and prostaglandin E_2_ (PGE_2_) expression, demonstrating osteocyte influence on osteoblast behavior in response to mechanical loading.^252^ A challenging but important aspect of these models is recreating realistic communication between joint compartments or cells, since this is a vital factor in both healthy and diseased joints. One approach involves the creation of microfluidic devices, such as “organ-on-chip,” that contain carefully designed channel and chamber systems to recreate tissue-level mass transport and fluid flow.^253^ For instance, a “multiorgan chip” has been developed to recapitulate the complexity of the human bone marrow niche. Results from experiments using this device show that it could be used to control colony formation of granulocytes, erythrocytes, macrophages, and megakaryocytes.^254^ Furthermore, it could be used to regulate the expression of osteopontin, VEGF, angiopoietin 1, and fibronectin in these cells with greater accuracy than in standard monolayer conditions. In a similar study, a novel microchip was used to apply dynamic hydraulic compression of 1 psi at 1 Hz to human bone marrow-derived MSCs, which controlled cell proliferation, differentiation, and increased osteogenic ECM production.^255^ These methodologies are likely to become ever more important as model systems are developed to incorporate more aspects of the osteochondral microenvironment from macroscale structure down to the level of mass transport and molecular diffusion. However, attaining that level of complexity will require significant research. In the interim, the use of explant (multitissue) systems is another tool in our research armamentarium, which can be used to replicate the complex interactions between osteochondral tissues, while still retaining the levels of control that are required in the laboratory setting.

## EXPLANT MODEL SYSTEMS

An alternative to 3D co-culture model system, which as we have seen can be complex, is *ex vivo* or explant model system. Explants are a valuable tool as they allow investigation of cell responses to controlled stimuli or environments while maintaining some aspects of the *in vivo* tissue, in particular the native ECM microenvironment. As OA is now recognized to be a disease of the whole joint, the use of osteochondral explants allows for the controlled analysis of how changes in one tissue type might affect another. Traditionally, femoral head or osteochondral core samples, from small or large animals, respectively, have been used for this purpose. These are of particular utility where the highly specialized ECM of bone or cartilage plays key roles in regulating cell behavior in response to specific stages or aspects of disease. While a potential drawback of using explants is that excessive ECM can limit diffusion, both bone and cartilage cells are unique in that they are specifically adapted for low oxygen and nutrient environments, facilitating the effective use of osteochondral explant culture. Furthermore, explants provide increased ability to control the mechanical and biochemical environment compared with *in vivo* experiments. These factors, among others, have seen an increased interest in the use of the explant model system in orthopedic research as well as many other areas of medicine.

To study the effects of physiological and pathological loading on skeletal tissues, such explant systems can be mechanically stimulated using modified mechanical/material testing protocols. A range of loading modes are available including unconfined compression, indentation, tension, and osmotic and hydrostatic pressures. The parameters that are used with these systems can test tissues responses to a variety of different types of loading and frequencies. For example, simple compression of bovine cartilage explants promotes matrix biosynthesis while dynamic (cyclic and intermittent) loading can differentially stimulate chondrocyte metabolism.^256^ Similarly, differences in cell and tissue responses to physiological and pathological loads can be studied using modifications of these test setups. For example, impact loading of cartilage explants, at different timepoints, showed a delayed (but significant) biological response following low impact, whereas high impact caused early and strong degenerative changes. The use of high impact (defined as imparting energy levels of 2.8 J) also resulted in a decreased tissue stiffness and increased cell death, which corelated with those degenerative changes. Furthermore, these changes were maintained for 4 weeks, and tissue degradation was manifested as increased glycosaminoglycan release and decreased overall content. These data provide consistent and realistic comparisons with real cases of joint injury and disease and demonstrate the utility of explants in this scenario.^257^ From a practical perspective, the ease with which biochemical agents can be added or detected in these mechanically stimulated culture environments is an additional advantage. For example, compression of cartilage explants was shown to modulate the proinflammatory responses that are normally generated in response to IL-1β and IL-4 stimulations.^258^

Explant cultures also have utility in determining the precise loading conditions and regimes that are most relevant to the joint and also how that initial damage can progress to disease. Using these systems, it was determined that applying compressive loading to cartilage explants at low frequency but for long time periods can produce a greater damage response than the same loads at higher frequency over shorter periods.^259^ Furthermore, close control of the precise magnitude of loading on cartilage explants (compression: 4–25 MPa) demonstrated force-specific apoptotic responses in chondrocytes peaking, as expected at higher magnitudes.^259^ More recently, this apoptotic response to high magnitude mechanical loading has been linked to increased mitochondrial dysfunction, including decreased basal respiration and ATP turnover, through direct mechanosignaling pathways.^260–262^ Similar investigations using bone explants have explored loading thresholds for eliciting tissue damage responses and to compare the micro- and macro-architecture of ovine, bovine, and human subchondral bone.^263–266^ Despite these insights, there remains much to learn about cell responses to loading in the joint, not least the order of cell response events following injury, the extent and importance of cellular crosstalk, and the loading conditions that lead to tissue microdamage. The expanding portfolio of well characterized explant models in the field has the potential to address many of these outstanding questions and provides an important preclinical tool in furthering our understanding of the joint injury and disease.

## SUMMARY AND FUTURE PERSPECTIVES

The complexity of the cellular, architectural, and mechanical environments of the joint means that a range of model systems are required to understand the processes underpinning its health and disease. Improving our understanding of these key processes is critical for the identification of potential therapeutic targets. For this, it is important that the correct experimental model is selected to allow interrogation of the relevant question. Research advances over the last decade, in diseases like OA, have made it clear that multiple interacting signaling pathways and cell types must be considered rather than isolated targeting of a single pathway or molecule. In particular, investigating the impact of mechanosignaling on OA-relevant signaling pathways is a rapidly expanding area in the field, but there remain technical challenges. As discussed above, different *in vitro* and *ex vivo* models have complementary advantages and can be leveraged to determine mechanisms behind the different physiological and pathological features of the system. In addition, microfluidic/joint-on-a-chip technologies and mechanically stimulated osteochondral explant models allow the examination of bone-cartilage crosstalk in tightly controlled culture environments, and these are likely to be key going forward. Central to the success of these technologies is their ability to accurately recreate *in vivo* interactions that occur in OA, which, in turn, will allow identification and refinement of drug targets within the joint. As use of advanced joint-on-a-chip and explant systems becomes more widespread, it is important that validation and comparison with model systems (particularly *in vivo* preclinical models) is carefully conducted. The increased throughput and precision of these osteochondral models will decrease the downstream failure rate of identified therapeutic targets and allow rapid and accurate screening of potential targets. In conclusion, a wide range of bone, cartilage, and osteochondral experimental models are now available to researchers in the field of musculoskeletal medicine. These approaches continue to be refined, and, their complexity increased, to incorporate more cell types, treatments, and stimuli. Recent advances in the development of mechanically stimulated higher-throughput models and explant cultures, along with biomaterials technology, are significantly impacting our understanding of OA pathology. In particular, the ability to create biomaterial environments with spatially controlled physical properties through additive manufacturing techniques, such as 3D printing, provides distinct opportunities for detailed investigation of cell responses and *in vitro* model fabrication. Furthermore, combining these advances with techniques such as biomaterial-mediated RNA interference will allow the controlled shutdown of signaling pathways involved in cell responses and identification of possible therapeutics. In the near future, this will lead to the development of more effective therapies and disease-modifying drugs for this complex disease.

## Data Availability

Data sharing is not applicable to this article as no new data were created or analyzed in this study.
